# Alemtuzumab in a Large Real-Life Cohort: Interim Baseline Data of the TREAT-MS Study

**DOI:** 10.3389/fneur.2021.620758

**Published:** 2021-08-05

**Authors:** Tjalf Ziemssen, Frank Hoffmann, Stephan Richter, Ulrich Engelmann, Robin White

**Affiliations:** ^1^Department of Neurology, Center of Clinical Neuroscience, Carl Gustav Carus University Hospital, Dresden, Germany; ^2^Klinik für Neurologie, Krankenhaus Martha-Maria Halle-Doelau, Halle (Saale), Germany; ^3^Zentrum für Neurologie und Psychiatrie, MIND, Stuttgart, Germany; ^4^Medical Affairs, Sanofi-Aventis Deutschland GmbH, Neu-Isenburg, Germany

**Keywords:** alemtuzumab, non-interventional study, risk-management plan, Germany, real world data, multiple sclerosis, effectiveness, safety

## Abstract

The non-interventional long-Term study foR obsErvAtion of Treatment with alemtuzumab in active relapsing–remitting MS (TREAT-MS) study collects the so far largest real-life cohort regarding utilization, long-term effectiveness, and safety of alemtuzumab, a humanized monoclonal antibody directed against the cell surface glycoprotein CD52, in adult patients with active relapsing–remitting multiple sclerosis (RRMS). An interim analysis of baseline parameters at inclusion of a non-interventional real-world study about alemtuzumab in Germany including previous multiple sclerosis (MS) medication utilization, MS activity, severity, and duration, as well as comorbidities was performed. Of the 883 patients, 71.6% were women. Mean age was 35.7 ± 9.2 years, time since first MS symptoms (=disease duration) is 8.0 ± 6.8 years, and Expanded Disability Status Scale (EDSS) is 2.7 ± 1.8 points (range, 0.0–7.5 points). The number of relapses in the 12 and 24 months prior to inclusion were 1.6 ± 1.2 and 2.2 ± 1.8, respectively. Of the patients, 14.4% were treatment naive, while for the majority, a wide spectrum of MS disease-modifying treatments (DMTs) and treatment sequences were documented. Overall, interferon beta (IFN-beta) was reported most frequently (52.4%), followed by fingolimod (35.2%), natalizumab (34.9%), and glatiramer acetate (28.9%). Patients with longer disease duration and higher EDSS had a higher number of previous DMTs. Compared to the pivotal phase 2/3 studies, RRMS patients starting alemtuzumab treatment had a longer disease duration in real-world conditions. There was variety of different treatment sequences before the final switch to alemtuzumab. In the future, linking these treatment sequences or other baseline characteristics with effectiveness and safety outcomes might be useful to support treatment decisions. Registered at Paul-Ehrlich-Institut under NIS 281.

## Introduction

The treatment landscape for multiple sclerosis (MS) has substantially changed, with the approval of more than 10 new drugs in the last decade. High-efficacy treatments appear to improve the long-term outcomes of MS patients (1) but are often only considered as second- or third-line options due to label restrictions or at the discretion of the treating physician. Two general treatment paradigms can be applied, either a maintenance-escalation approach, where a medication is given continuously and patients are switched to a higher efficacy drug upon disease activity, or a pulsed immune reconstitution therapy, which involves few treatment pulses with long intermittent treatment-free phases (2). Alemtuzumab (Lemtrada®, Sanofi Genzyme) is given as a pulsed immune reconstitution therapy in usually two treatment phases, which leads to sustained and treatment-free effectiveness (3, 4). Alemtuzumab is a humanized monoclonoal IgG1kappa-type antibody binding to the cell surface protein CD52, which is expressed in large amounts on B and T lymphocytes (5). After binding of alemtuzumab to CD52, circulating lymphocytes are depleted either by complement-induced or antibody-dependent cell-mediated cytotoxicity (6). After depletion, B- and T-lymphocyte repopulation occurs in a defined pattern and has demonstrated beneficial long-term effects (7).

Overall, alemtuzumab appears to reprogram the immune repertoire, which manifests in the special kinetics of immune cell populations, the increased production of antiinflammatory cytokines, and the very long duration of action (8). Three randomized, rater-blinded clinical trials assessing the efficacy of alemtuzumab in MS treatment, using an effective comparator drug, have been performed: CAMMS223 (9), CARE-MS I (10), and CARE-MS II (11). In sum, alemtuzumab significantly reduced clinical and radiological disease activity and slowed down progression of relapsing–remitting MS (RRMS) to secondary progressive MS, also in the long-term and in patients with highly active disease (HAD) (4, 12–15).

In the European Union, in 2013, alemtuzumab has been marketed as a treatment for RRMS with active disease defined by clinical or imaging features. In the USA, in 2014, the drug has been approved for RRMS and progressive–relapsing MS treatment but only for patients who did not have a satisfying response to two or more drugs (16) (i.e., for third-line therapy). In 2019, alemtuzumab has undergone a procedure under Article 20 of Regulation (EC) No 726/2004 resulting from pharmacovigilance data, which led to label change effective January 2020 (17). Alemtuzumab should now only be used to treat RRMS if the disease is highly active despite treatment with at least one disease-modifying therapy or if the disease is worsening rapidly (18). Alemtuzumab must also no longer be used in patients with certain heart, circulation, or bleeding disorders or in patients who have autoimmune disorders other than multiple sclerosis.

Data on the utilization and the treatment outcomes of alemtuzumab in the real-world clinical practice are limited to few reports on small, mostly monocentric cohorts (19, 20) or a retrospective data collection, respectively (21). There is a need for high-quality, comprehensive, and valid real-life evidence data, as these data cover additional aspects of patient care and expand the data available by complementary information (22, 23).

The aim of the non-interventional long-Term study foR obsErvAtion of Treatment with alemtuzumab in active relapsing–remitting MS (TREAT-MS) study is to establish a broader real-world database on the utilization and effectiveness, safety, and other aspects of the drug in everyday clinical practice in Germany (24). The current interim analysis describes the cohort of patients before the alemtuzumab label change with particular focus on the treatment profile, disease characteristics, and comorbidities before alemtuzumab start.

## Design and Methods

### Design

TREAT-MS is a prospective and retrospective, multicenter, open-label, non-interventional long-term study that collects data from neurologists in specialized MS centers (clinics or outpatient departments) in Germany (24). The study was registered in a publicly accessible database at Paul-Ehrlich Institute (regulatory authority) under NIS 281.

### Patients

Patients are eligible for documentation if they are newly treated with alemtuzumab or have initiated treatment earlier and are followed up on the long term.

### Study Flow and Parameters

Study parameters include the following: demographics, comorbidities, MS anamnesis and characteristics including relapses over time, Expanded Disability Status Scale (EDSS), lesions on MRI, and as patient-related outcomes, Symbol Digit Modality Test (SDMT), Patient-Reported Indices for MS (PRIM US), EuroQol 5D-3L, and Work Productivity and Activity Impairment Questionnaire (WPAI) (25). The Clinical Global Impression-Severity (CGI-S) test expresses the experience-based impression of the treating physician on the severity of the patient's illness in a 7-point scale (26). Analogously, the CGI-S can be determined by the patient to show the evaluation of the patient on his or her clinical condition.

### Treatment

Alemtuzumab is administered as two annual courses (on 5 consecutive days at baseline and on 3 consecutive days 12 months later), and patients are followed up for safety as per local labeling. Patients could receive up to two additional courses (12 mg/day ×3 days) ≥12 months after the most recent course or treatment with other DMTs as needed.

Neurologists and MS nurses were guided by the MS documentation system for physician, nurse, and patient (MSDS 3D) Lemtrada-TREAT-MS module through the entire management of treatment, including monitoring of the first and second infusion courses, necessary examinations, and regular laboratory screenings (27, 28).

Statistical analyses were performed in an exploratory manner using descriptive statistical methods. For continuous variables, the number of patients with non-missing and missing data, mean, standard deviation, minimum, 25% quantile, median, 75% quantile, and maximum were calculated. For ordinal and categorical variables, frequencies were calculated. Incomplete data sets were included in the analysis. Imputations were only done for missing dates for days (substituted by the 15) and for months (substituted by June), while years were not substituted. Given the descriptive character of the study, no further imputations were deemed appropriate.

No sensitivity analyses were done.

A treatment pathway is defined as a unique longitudinal sequence of discrete MS treatments [disease-modifying therapies (DMT)] and is differentiated based on introduction of discrete DMTs in patients' MS treatment course. Treatment pathways were visualized in Sankey diagrams, generated through SAS, version 9.4 (SAS Institute Inc., Cary, NC, USA) (29). As another visualization approach, scatterplots were generated based on Multiple Sclerosis Severity Score (MSSS), which relates scores on the EDSS to the distribution of disability in patients with comparable disease durations (30).

The total cohort at the data cutoff date February 10, 2020 comprised 883 patients. Statistical analyses were done with IBM SPSS for Windows, Version 15.0.0.

## Results

### Setting

Of all physicians who contributed at least one eligible patient for the present analysis, 41 (34.7%) were hospital-based and 77 (65.3%) were resident neurologists. Data from 426 (48.2%) and 457 (51.8%) patients were documented by hospital-based and resident neurologists, respectively.

### Baseline Characteristics of Patients

**Figure 1:**
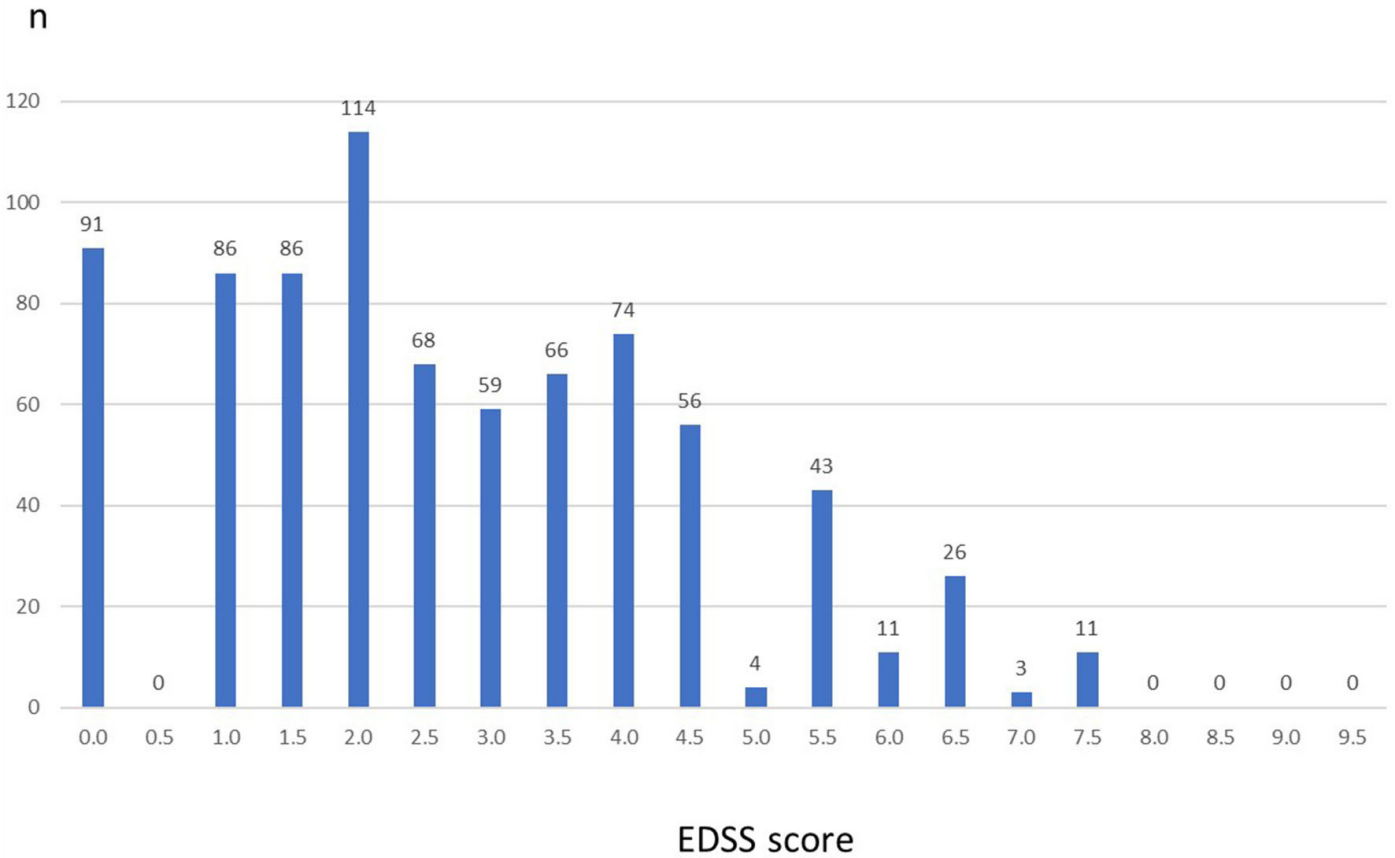
Expanded Disability Status Scale (EDSS) at baseline (*n* = 883). Columns represent number of patients for each EDSS value. There were no patients with values above 7.5.

Baseline characteristics of the 883 patients are summarized in Table 1. Mean age at baseline was 35.7 ± 9.2 years (range, 16–63 years). The majority (71.6%) were female. Mean time since first MS symptoms (=disease duration) was 8.0 ± 6.8 years and since MS diagnosis was 7.2 ± 6.3 years. The median EDSS was 2.5, with a range from 0.0 to 7.5. While 63.2% of the patients had an EDSS ≤ 3, 36.8% had a baseline EDSS >3. Figure 1 displays the distribution of EDSS categories. The mean number of relapses in the 12/24 months prior to inclusion was 1.6 ± 1.2/2.2 ± 1.8. Clinical Global Impression (CGI) assessed by the physician or patients at inclusion assessment was 4.8 ± 2.7 and 3.2 ± 1.7, respectively.

**Table 1:** Baseline characteristics.

**Variable**	**Total cohort**	
	***N***	**Value**
Age (years)	883	35.7 ± 9.2
Range		16–63
Sex, Female, %	632	71.6
Male, %	251	28.4
**Multiple sclerosis characteristics**		
Time (years) since first MS symptoms until inclusion into study	668	8.0 ± 6.8
Time (years) since MS diagnosis until inclusion into study	793	7.2 ± 6.3
RRMS, %	823	95.4
**Relapses during last 12 months before inclusion into study, %**		
0	127	16.3
1	295	37.8
2	221	28.3
3	87	11.1
Missing	102	
Mean ± SD	781	1.6 ± 1.2
**Relapses during last 24 months before inclusion into study, %**		
0	89	12.7
1	187	26.6
2	182	25.9
3	128	18.2
Missing	181	
Mean ± SD	702	2.2 ± 1.8
**Magnetic resonance imaging**		
Contrast medium enhancing lesions present at 1st pretreatment visit, %	397	54.7
**Gd+** **lesions**		
0	226	31.4
1	69	9.6
2	56	7.8
3+	96	13.3
**T2 lesions**		
0	38	4.8
1	16	2.0
2	10	1.3
3+	174	21.8
EDSS total	798	2.7 ± 1.8
≤ 3	504	63.2
>3	294	36.8

*EDSS, Expanded Disability Status Scale, RRMS, relapsing–remitting multiple sclerosis.*

*Values are percentages or means ± standard deviation (SD)*.

### MS Pre-treatment With DMT

About every seventh patient (*n* = 127; 14.4%) was treatment naive. In contrast, 722 (81.7%) had received any DMT (3.9% unknown). In detail, 21.7, 30.4, 18.5, 9.5, and 2.3% had received one, two, three, four, or five or more pretreatments with MS medications, respectively.

The MS treatment history before the initiation of alemtuzumab is listed by decreasing frequency in Table 2. Interferon-beta (IFN-beta) was reported most frequently (52.4%), followed by fingolimod (35.2%), natalizumab (34.9%), and glatiramer acetate (28.9%). With regard to the last MS medication before alemtuzumab initiation, 22.0% received fingolimod, 14.8% natalizumab, and 8.6% IFN-beta therapy.

**Table 2:** Disease-modifying treatments (DMTs) pretreatment.

**DMT pre-treatment**	**Total (*N* = 883)** ***n* (%)**
Total	727 (82.3)
Interferon-beta	463 (52.4)
Fingolimod	311 (35.2)
Natalizumab	308 (34.9)
Glatiramer acetate	255 (28.9)
Other^a^	131 (14.8)
Dimethyl fumarate	109 (12.3)
Teriflunomide	44 (5.0)
Mitoxantrone	18 (2.0)
Azathioprine	9 (1.0)
Unknown	5 (0.6)
Methotrexate	2 (0.2)
Rituximab	1 (0.1)

*Values are n and percentages of total.*

a *“Other” includes unspecified drugs in 31 patients, daclizumab in 20 patients, immunoglobulins in six patients, and a variety of other drugs in the remaining patients*.

### Characterization of the Disease Status at Baseline

The EDSS, the duration since initial MS symptoms, and the number of MS relapses in the previous year are useful parameters to evaluate the disease status. In order to visualize these parameters and to relate them to the number of previous MS medications, scatterplots were used combining the parameters. This allows an evaluation of the MS disease status of TREAT-MS alemtuzumab-treated patients at baseline.

**Figure 2:**
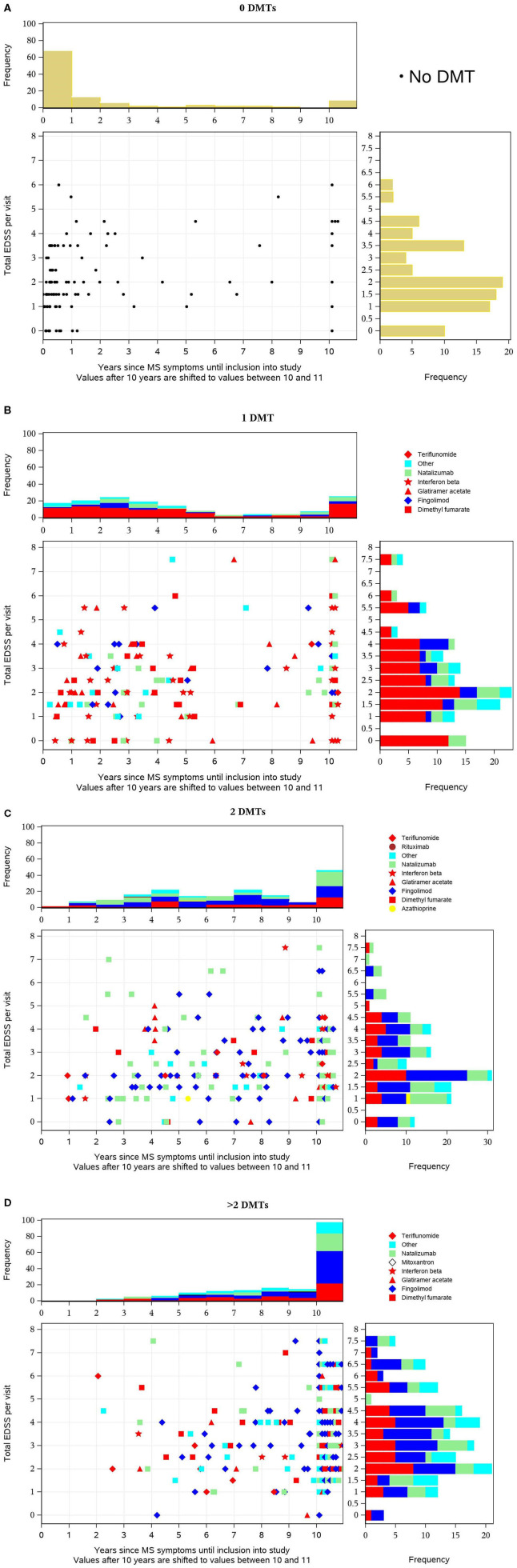
**(A–D)** Distribution of Expanded Disability Status Scale (EDSS) values (y-axis) vs. disease duration before inclusion into the study (x-axis) by disease-modifying treatment (DMT) pretreatment. Each dot in the diagram represents one patient's value in relation to both parameters at baseline. For pretreated patients, the most recent DMT is distinguished by different symbols as indicated in the legend and the basic therapies glatiramer acetate, dimethyl fumarate (DMF), interferon-beta, and teriflunomide are colored red so that they can be easily distinguished from escalation therapies. The horizontal bars on the right show the distribution (histogram with frequencies in percent) of EDSS values, the vertical bars above the scatterplot the distribution of time intervals across all patients (percentage).

The scatterplots (Figures 2A–D) show the distribution of EDSS values (y-axis) vs. disease duration (years before inclusion into the study, x-axis) by DMT pretreatment and described in detail in the figure legend.

**Figure 3:**
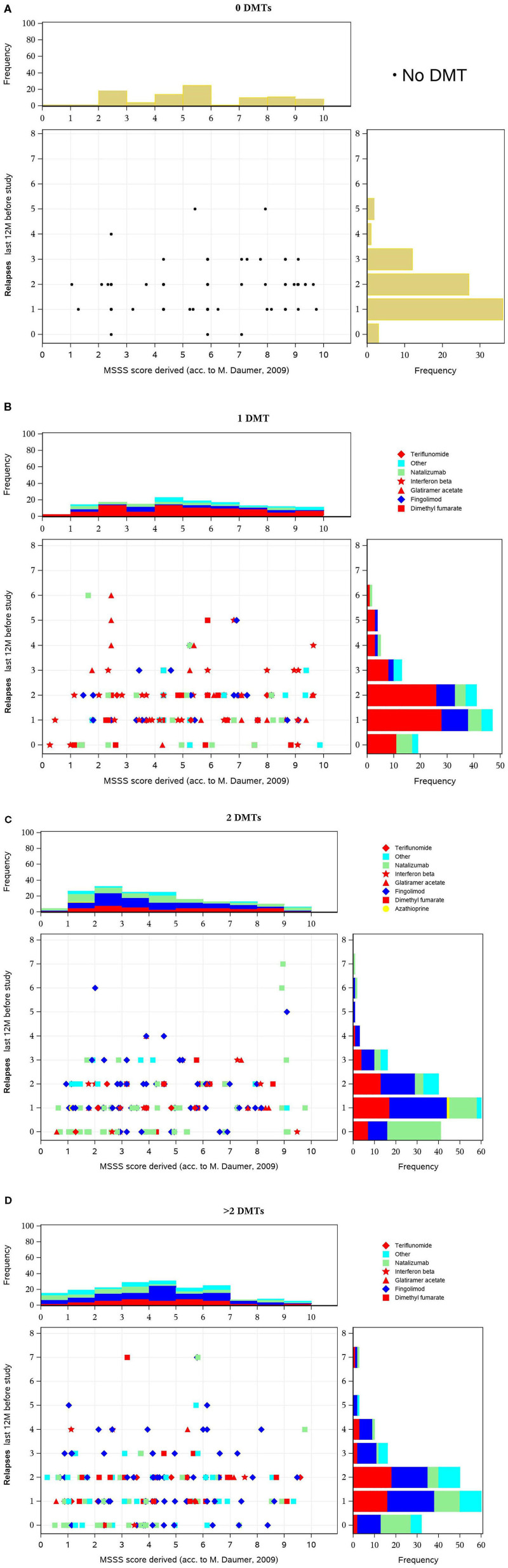
**(A–D)** Multiple Sclerosis Severity Score (MSSS) (y-axis) vs. number of exacerbations (x-axis) in the 12 months before inclusion, by disease-modifying treatment (DMT) pretreatment. These figures display scatterplots of MSSS (x-axis) vs. number of relapses (y axis) in the 12 months before inclusion, in patients with no, one, two, or more DMT at baseline. In all subgroups, there was a peak of one or two relapses and a similar distribution with a predominance of lower MSSS values. There is no distinct pattern of medication use in the various groups. However, escalation therapy is more often used in patients with a higher number of disease-modifying treatments (DMTs).

Each dot in the diagram represents one patient's value in relation to both parameters at baseline. For pretreated patients, the most recent DMT is distinguished by different symbols as indicated in the legend, and the basic therapies glatiramer acetate, dimethyl fumarate (DMF), interferon-beta, and teriflunomide are colored red so that they can be easily distinguished from escalation therapies. The horizontal bars on the right show the distribution (histogram with frequencies in percent) of EDSS values and the vertical bars above the scatterplot the distribution of time intervals across all patients (percentage). In treatment-naive patients, the majority had a short disease duration (in two-thirds of patients <1 year before inclusion) and were predominantly in the lower EDSS categories (with peaks at 0–2 and 3.5) (Figure 3A). In patients who previously received one DMT, the EDSS pattern does not differ much. In contrast, the time pattern does, since peaks occur 2 years after diagnosis and after 10+ years. The distribution of the various DMTs appears similar across the different EDSS and the different time periods, respectively. The majority of patients received baseline therapies as indicated by the red color (Figure 3B). In patients previously treated with two DMTs, a trend to higher EDSS values is visible. Furthermore, the proportion of patients with long disease duration (10+ years) is nearly at 50%. Fewer patients are on interferon beta and more are on fingolimod, natalizumab, and other DMTs. Overall, the proportion of basic therapies (red) was lower compared to patients with one DMT (Figure 3C). In patients pretreated with three or more DMTs, the described changes are even more pronounced: compared to the previously described subgroups, a higher proportion of patients have a higher EDSS value, and nearly 90% had a disease duration of 10+ years (Figure 3D).

The MSSS combines EDSS and disease duration and is considered to be a powerful method for comparing disease progression using single assessment data (30). The score predicts disease severity over time (31). Figures 3A–D show the scatterplot of MSSS vs. number of relapses in the 12 months before inclusion, in patients with no, one, two, or more DMTs at baseline. In all subgroups, there was a peak of one or two relapses and a similar distribution with a predominance of lower MSSS values. There is no distinct pattern of medication use in the various groups. However, escalation therapy is more often used in patients with a higher number of DMTs.

### Visualization of Treatment Pathways

Among the pretreated patients, 214 different treatment sequences were documented. Table 3 shows the 15 most frequent pretreatments and pathways in descending order.

**Table 3:** Most frequent treatment pathways prior to switch to alemtuzumab.

**DMT pre-treatment**			***n* Patients**	**% Of total (*N* = 886)**
**First**	**Second**	**Third**		
Interferon-beta			52	5.9
Interferon-beta	Fingolimod		42	4.7
Interferon-beta	Natalizumab		36	4.1
Natalizumab			28	3.2
Dimethyl fumarate			26	2.9
Interferon-beta	Natalizumab	Fingolimod	24	2.7
Fingolimod			23	2.6
Glatiramer acetate			23	2.6
Other			17	1.9
Glatiramer acetate	Fingolimod		13	1.5
Interferon-beta	Interferon-beta	Fingolimod	11	1.2
Interferon-beta	Glatiramer acetate	Fingolimod	10	1.1
Interferon-beta	Glatiramer acetate		10	1.1
Interferon-beta	Dimethyl fumarate		9	1.0
Glatiramer acetate	Natalizumab		9	1.0

*In line with three DMTs, patients received the named three drugs in the order first–second–third before switching to alemtuzumab.*

*Values are n and percentages of total*.

**Figure 4:**
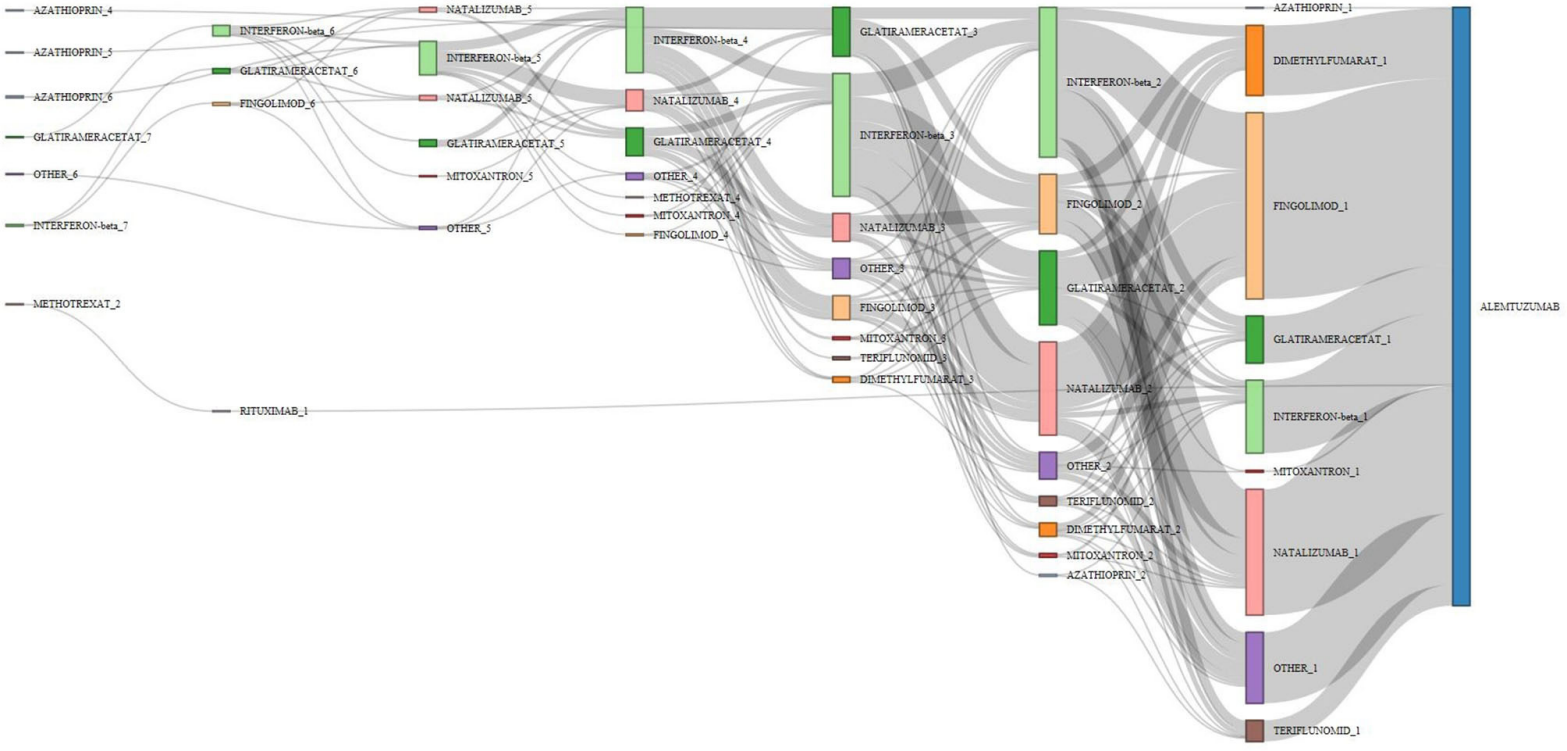
Pathways of disease-modifying treatments (DMTs) before final switch to alemtuzumab. The figure displays the pathways of DMTs before the final switch to alemtuzumab (before or at the inclusion visit of the study). The various MS medications are represented by different colors (alemtuzumab blue, fingolimod light orange, interferon-beta light green, glatiramer acetate green); the height of the stacked vertical bar represents the number of patients treated with the respective MS medication. The width of the lines (ribbons) that connect the individual stacked columns visualizes the number of patients who are transferred to the same (same color) or another medication (different color). It is clearly visible by the wide ribbons that “typical” pathways in this study were from interferon-beta to natalizumab, from interferon-beta to fingolimod, and from natalizumab to fingolimod. Patients were excluded if the exact treatment order could not be determined (29 cases unknown, 127 no pretreatment, 104 last pretreatments not identifiable), leaving 623 patients in the chart. Chronological treatment sequence order starts on the left and ends with alemtuzumab on the right. See also editable Figure 5.

Duration of previous therapy was reported in 55% of patients. Among these, in <5%, duration was <3 months. Figure 4 visualizes the main treatment pathways, which finally end up in 623 (pretreated) patients displayed in the blue alemtuzumab column on the right.

**Figure 5:**
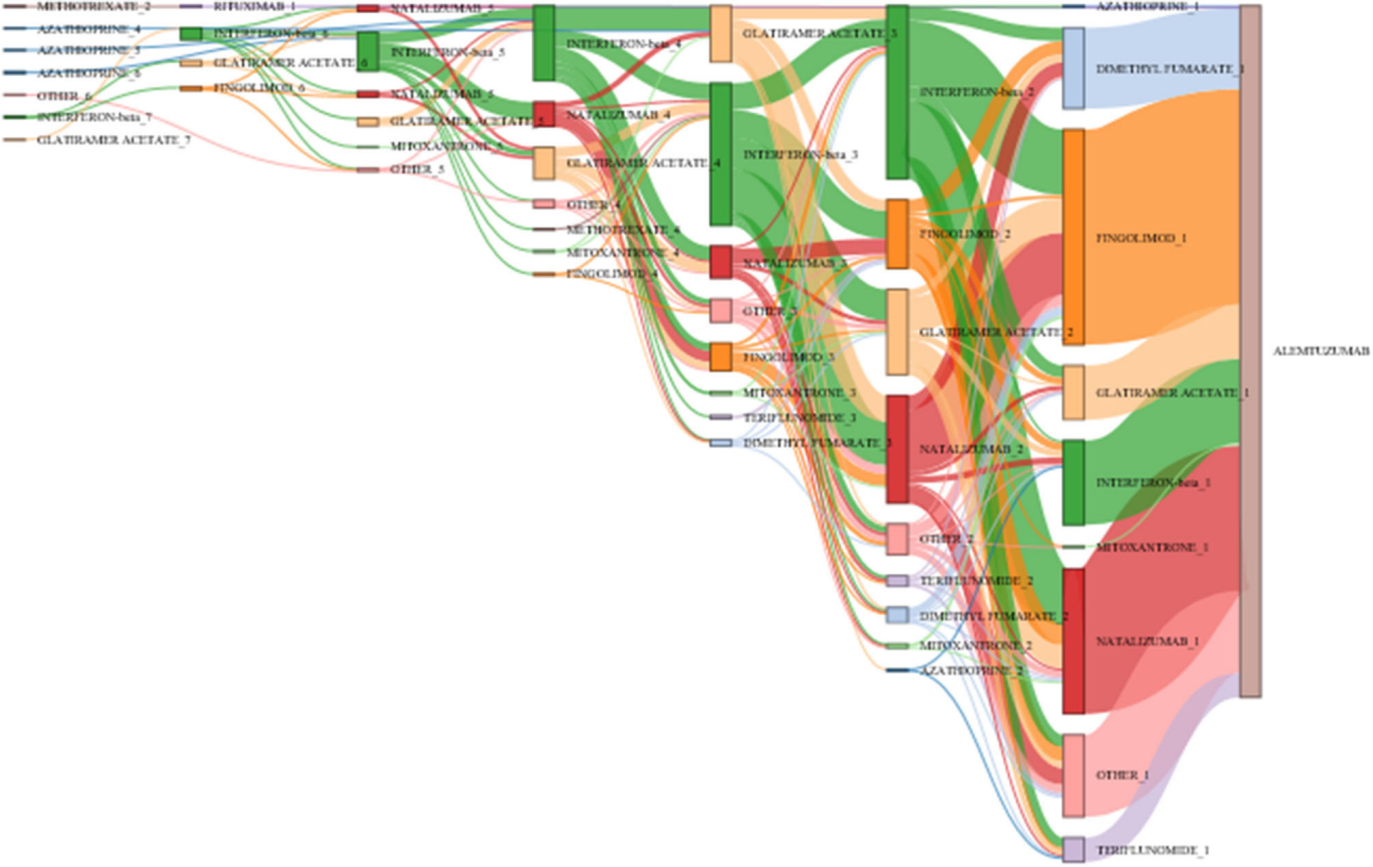
Pathways of disease-modifying treatments (DMTs) before final switch to alemtuzumab. The figure illustrates the various treatment pathways as in Figure 4. By moving the cursor over the connecting ribbons, the respective number of patients treated with the previous DMT are indicated. The number following the DMT name indicates the position in the treatment sequence. The bars can be moved vertically to change the view of the Sankey plot.

Figure 5 shows the same plot in HTML format in which the cursor roll-over the connecting ribbon will indicate the respective number of DMTs and the sequence number before alemtuzumab. The nodes can also be shifted vertically to change the view of the Sankey plot.

### Concomitant Diseases

Concomitant diseases at baseline were reported in 30.0% of patients (Table 4). The System Organ Classes that were most frequently affected were psychiatric disorders (11.6%), metabolism and nutrition disorders (10.0%), and immune system disorders (4.6%). The latter comprised mostly allergies but also one case of autoimmune disorder. As relevant disease (which prevent therapy as specified in the latest update of the Lemtrada® SmPC in January 2020), thyroid diseases were named in 31 cases, nephropathy in 2 cases, and immune thrombocytopenic purpura (ITP) in 1 case (Table 5).

**Table 4:** Concomitant disease by system organ class.

**SOC**	**Total** **(*N* = 883)** ***n***	**%**
Any disease	344	39.0
Blood and lymphatic system disorders	12	1.4
Cardiac disorders	13	1.5
Congenital, familial, and genetic disorders	24	2.7
Ear and labyrinth disorders	5	0.6
Endocrine disorders	18	2.0
Eye disorders	29	3.3
Gastrointestinal disorders	25	2.8
General disorders and administration site conditions	18	2.0
Hepatobiliary disorders	7	0.8
Immune system disorders	41	4.6
Infections and infestations	28	3.2
Injury, poisoning and procedural complications	14	1.6
Investigations	13	1.5
Metabolism and nutrition disorders	88	10.0
Musculoskeletal and connective tissue disorders	43	4.9
Neoplasms benign, malignant, and unspecified	14	1.6
Nervous system disorders	114	12.9
Pregnancy, puerperium, and perinatal conditions	1	0.1
Psychiatric disorders	102	11.6
Renal and urinary disorders	30	3.4
Reproductive system and breast disorders	10	1.1
Respiratory, thoracic, and mediastinal disorders	31	3.5
Skin and subcutaneous tissue disorders	28	3.2
Surgical and medical procedures	28	3.2
Vascular disorders	45	5.1

*SOC, system organ class (from the Medical Dictionary for Regulatory Activities)*.

**Table 5:** Diseases of particular interest.

**Disease**	***n***	**% Of total** **(*N* = 883)**
Immune thrombocytopenic purpura	1	0.1
Nephropathy	2	0.2
**Thyroid diseases**		
Hypothyroidism	53	6.2
Hyperthyroidism	10	1.2
Hashimoto's thyroiditis	11	1.3
Graves' disease (Basedow)	2	0.2
Other	12	1.4

*Values are n and percentages of total*.

There were no patients with history of angina pectoris, myocardial infarction, or stroke at baseline.

## Discussion

The present analysis focused on the detailed characterization of MS patients who, irrespective of the type of prior treatment and the MS duration, are finally treated with alemtuzumab. The data complement the body of evidence from 1,500 patients that received alemtuzumab in the randomized controlled trials [CAMMS223 (9), CARE-MS I (10), and CARE-MS II (11)].

Compared to the initial alemtuzumab registration studies, the treament landscape and armamentarium of drugs have substantially changed, which needs to be considered in the interpretation of results. Compared with the baseline characteristics from the pivotal CARE-MS I and CARE-MS II trials, patients in TREAT-MS at enrollment had a comparable mean duration of disease since first symptoms (CARE-MS I, 2.1 years; CARE-MS II, 4.5 years; TREAT-MS, 3.4 years), a higher percentage with EDSS score >3 (CARE-MS I, 2%; CARE-MS II, 31%; TREAT-MS, 37%), a higher percentage who received treatment with fingolimod (only introduced in 2011: CARE-MS I and II, 0%; TREAT-MS, 35%) or natalizumab (CARE-MS I, 0%; CARE-MS II, 4%; TREAT-MS, 35%) prior to enrollment. They tended to have similar relapse activity in the 2 years before alemtuzumab treatment initiation. Furthermore, in TREAT-MS, the sex and age distribution at baseline was similar to the two registration studies. Generally, patients with more advanced MS are treated with alemtuzumab under clinical practice conditions in Germany. However, every seventh patient was treatment naive prior to alemtuzumab initiation.

In line with the many treatment options for MS patients available today, a great variety of pretreatment patterns were documented. The Sankey diagram visualizes this diversity, over time and across DMTs. Few typical patterns emerged, with switches from IFN-beta to natalizumab or fingolimod and from natalizumab to fingolimod being the most eminent ones.

The relatively high number of patients recruited from centers in all parts of the country and different types of centers (51.8% resident neurologists, 48.2% from various types and sizes of hospitals) is a strength of the study. It describes “typical” alemtuzumab patients as treated under real-life conditions; however, physicians may have assigned patients to the study based on the severity of their disease, on the observation that they did not respond well to other drugs, or the presence of complex comorbidities. These factors might lead to a non-representative study population.

Based on the assessment of the periodic safety update report (PSUSA) for alemtuzumab, in 2020, contraindications were added to the SmPC, in particular relating to cardiovascular disease (including history of stroke, angina pectoris, and myocardial infraction) and concomitant autoimmune diseases besides MS (32). While no patients had the named cardiovascular disease and only few had autoimmune diseases at baseline, the results of the present cohort will be an important contribution to the alemtuzumab safety database.

In conclusion, the present analysis revealed a broad variety of different treatment sequences before the final switch to alemtuzumab. In comparison to the pivotal phase 2 and 3 studies, RRMS patients starting alemtuzumab treatment had a longer disease duration in real-world conditions.

Recently, a dual-center retrospective study from Germany in 170 patients treated with alemtuzumab (PROGRAM^MS^) described the pretreatment (35 none, 52 basic, 50 natalizumab, 33 fingolimod) and found differences in treatment responses based on the previous use of DMT (33). In the future, linking treatment sequences or other baseline characteristics with effectiveness and safety outcomes might be useful to support treatment decisions (34, 35).

## Data Availability Statement

The datasets generated and/or analyzed during the current study are available from the corresponding author on reasonable request.

## Ethics Statement

The studies involving human participants were reviewed and approved by Ethikkommission der Universitäsklinik Dresden. The patients/participants provided their written informed consent to participate in this study.

## Author Contributions

TZ and UE developed the study design. FH, SR, and RW participated in the design of the study and contributed to the interpretation of results and the manuscript. RW initiated the drafting of the report and wrote the manuscript. All authors read and approved the final version of this manuscript.

## Conflict of Interest

RW and UE are employees of Sanofi. TZ received consulting and/or speaking fees (Almirall, Bayer, Biogen, Merck, Novartis, Roche, Sanofi, and Teva) and grant/research support (Biogen, Novartis, Sanofi, and Teva). FH received consulting and/or speaking fees (Alexion, Bayer, Biogen, CSL Behring, DIAMED Medizintechnik, Grifols, Ipsen, Merck, Novartis, Roche, Sanofi and Teva) and grant/research support (Bayer, Biogen, Merck, Novartis). SR received consulting and/or speaking fees (Biogen, Merck, Novartis, Roche, Sanofi) and grant/research support (Biogen, Merck, Celgene). The authors declare that this study received funding from Sanofi-Aventis Deutschland GmbH. The funder was involved in the study design, analysis, interpretation of data, the writing of this article and the decision to submit it for publication.

## Publisher's Note

All claims expressed in this article are solely those of the authors and do not necessarily represent those of their affiliated organizations, or those of the publisher, the editors and the reviewers. Any product that may be evaluated in this article, or claim that may be made by its manufacturer, is not guaranteed or endorsed by the publisher.
